# The Burden of Sepsis and Septic Shock in the Intensive Care Unit

**DOI:** 10.3390/jcm14196691

**Published:** 2025-09-23

**Authors:** Luigi La Via, Antonino Maniaci, Mario Lentini, Giuseppe Cuttone, Salvatore Ronsivalle, Simona Tutino, Francesca Maria Rubulotta, Giuseppe Nunnari, Andrea Marino

**Affiliations:** 1 Department of Anesthesia and Intensive Care, University Hospital Policlinico “G. Rodolico-San Marco”, 95123 Catania, Italy; francesca.rubulotta@unict.it; 2 Department of Medicine and Surgery, University of Enna “Kore”, 94100 Enna, Italy; antonino.maniaci@unikore.it (A.M.); mario.lentini@unikore.it (M.L.); salvatore.ronsivalle@unikore.it (S.R.); 3 Department of Surgery, ASP 7, 97100 Ragusa, Italy; 4 Anesthesia and Intensive Care Unit, ‘Abele Ajello’ Hospital, ASP Trapani, 91026 Mazara del Vallo, Italy; giuseppe.cuttone@hotmail.it; 5 Department of Anesthesia and Intensive Care, Hospital “Giovanni Paolo II”, ASP 7, 97100 Ragusa, Italy; simona.tutino.29.9.92@gmail.com; 6 Department of General Surgery and Medical-Surgical Specialties, University of Catania, 95100 Catania, Italy; 7 Unit of Infectious Diseases, ARNAS Garibaldi Hospital, 95122 Catania, Italy; giuseppe.nunnari1@unict.it (G.N.); andrea.marino@unict.it (A.M.); 8 Department of Clinical and Experimental Medicine, University of Catania, 95100 Catania, Italy

**Keywords:** sepsis, intensive care unit, diagnosis, management, post-intensive care syndrome, ICU, septic shock, critical care

## Abstract

This narrative review synthesizes our current understanding of sepsis and septic shock burden in intensive care units (ICUs) worldwide. Based on a comprehensive but non-systematic literature search from 2000 to 2025, this review synthesizes our current understanding across eight key domains: epidemiology, pathophysiology, diagnostics, management strategies, long-term outcomes, disparities, and future directions. The global burden of sepsis, especially in the developed and developing world, is great: over 48 million cases per year, with mortality rates at the ICU level in the range of 30 to 50%, depending on geography and resources. The pathophysiological progression from an initial hyper-inflammatory state to immune paralysis underlies organ failure and complicates therapeutic targeting. Diagnostic approaches, including clinical scoring systems, biomarkers (e.g., procalcitonin, MR-proADM), and emerging AI tools, offer improved early detection but face challenges in reliability and accessibility. Management in the ICU remains anchored in timely antimicrobial administration, hemodynamic stabilization with balanced fluids and vasopressors, source control, and organ support, including lung-protective ventilation and kidney replacement therapy. Novel adjuncts, such as immunomodulators and extracorporeal therapies, show promise but demand further validation. Importantly, survivors face significant long-term sequelae—post-intensive care syndrome (PICS)—encompassing physical, cognitive, and psychological impairments, which require structured rehabilitation and follow-up. The future of sepsis care lies in integrating precision medicine—through molecular diagnostics, individualized immunotherapy, and AI-supported monitoring—with scalable, equitable implementation strategies that bridge the gap between high- and low-income settings. Addressing disparities and expanding rehabilitation services are essential to improving survival and long-term quality of life in sepsis survivors.

## 1. Introduction

Sepsis—a life-threatening organ dysfunction triggered by a dysregulated host response to infection—remains a global public health crisis, particularly in intensive care units (ICUs), where patients are most vulnerable. Global estimates from the 2017 Global Burden of Disease (GBD) study indicate approximately 48.9 million incident sepsis cases and 11 million sepsis-related deaths worldwide annually, accounting for nearly 20% of all global mortality [1,2,3]. While age-standardized sepsis incidence and associated mortality declined between 1990 and 2017 by 37% and 53%, respectively [2], these improvements are unevenly distributed, with sepsis continuing to disproportionately affect low- and middle-income countries (LMICs), children under five, and marginalized populations [2,4]. When examining ICU-specific populations, the burden intensifies considerably. A systematic review by Fleischmann-Struzek et al. (2020) found that among ICU-treated sepsis patients specifically, the incidence was 58 per 100,000 person-years, with hospital discharge mortality reaching 41.9%—substantially higher than the 26.7% mortality observed across all hospital-treated sepsis patients (including non-ICU settings) [2]. Additional meta-analyses focusing exclusively on ICU cohorts report even greater variation: ICU prevalence of sepsis at approximately 31% with associated ICU mortality rates ranging from 30% to 46%, depending on geographic region, study design, and severity assessment criteria [5,6]. These disparate figures underscore not only sepsis’s devastating impact in critical care settings but also the persistent methodological heterogeneity in how sepsis outcomes are measured and reported across different healthcare contexts and timeframes. In broader hospital-treated cohorts, the pooled mortality is estimated at 26.7% [5]. Region-specific studies further underscore significant variability: 30-day sepsis mortality averages ~24%, with ranges from 3% to nearly 47%, and 90-day mortality averaging ~32%, again with wide regional and study design-dependent variation [6]. These sobering statistics reflect not only the clinical severity of sepsis but also the complexity surrounding its recognition and management. The pathophysiological spectrum—from early immune activation and inflammatory cascades to subsequent immune suppression and multi-organ dysfunction—complicates timely diagnosis and amplifies the risk of adverse outcomes [2,7]. Diagnostic delays, variations in resource availability, and inconsistent implementation of evidence-based protocols (e.g., early antibiotics, fluid resuscitation, and bundle strategies) further contribute to suboptimal outcomes, even in high-income settings. In LMICs, these challenges are often exacerbated by limited infrastructure, scant epidemiologic surveillance, and delayed access to care. Given the complexity and heterogeneity in the sepsis literature, this narrative review aims to critically synthesize our current knowledge of the burden of sepsis and septic shock in ICU settings. Unlike systematic reviews that follow strict methodological protocols, this narrative approach allows for a broader integration of diverse evidence types while acknowledging the inherent limitations of selective literature inclusion.

## 2. Materials and Methods

### 2.1. Literature Search Strategy

This narrative review was conducted to provide a comprehensive synthesis of sepsis and septic shock burden in ICU settings. Unlike systematic reviews that follow PRISMA guidelines with predefined inclusion/exclusion criteria, this narrative approach allowed for flexible integration of diverse evidence types, including landmark studies, recent advances, and evolving concepts. This methodology was chosen to accommodate the heterogeneous nature of the sepsis literature and to provide a clinical context that strict systematic approaches might overlook. A comprehensive literature search was performed in PubMed/MEDLINE, Embase, Scopus, and the Cochrane Library from January 2000 to July 2025. The search terms included combinations of “sepsis”, “septic shock”, “intensive care unit”, “critical care”, “burden”, “epidemiology”, “mortality”, “management”, and “outcomes”, using both MeSH terms and free-text keywords.

### 2.2. Selection Criteria

We included peer-reviewed studies, systematic reviews, meta-analyses, clinical guidelines, and high-quality observational reports that addressed epidemiology, pathophysiology, diagnosis, treatment, or outcomes of sepsis and septic shock in adult ICU populations. Pediatric studies, animal experiments, case reports, conference abstracts, and non-English publications were excluded. The retrieved studies were screened for methodological rigor, relevance, and applicability to ICU settings. Whenever available, we prioritized large-scale, multicenter, and high-quality prospective data. For controversial or evolving topics, contrasting findings were presented and critically analyzed to highlight strengths, limitations, and implications for clinical practice. Data were extracted independently by two reviewers and summarized narratively, given the heterogeneity in study designs, patient populations, and outcome measures. Quantitative data are reported descriptively, with emphasis on trends, magnitude of effects, and clinically meaningful endpoints.

### 2.3. Literature Selection Process

**Figure 1:**
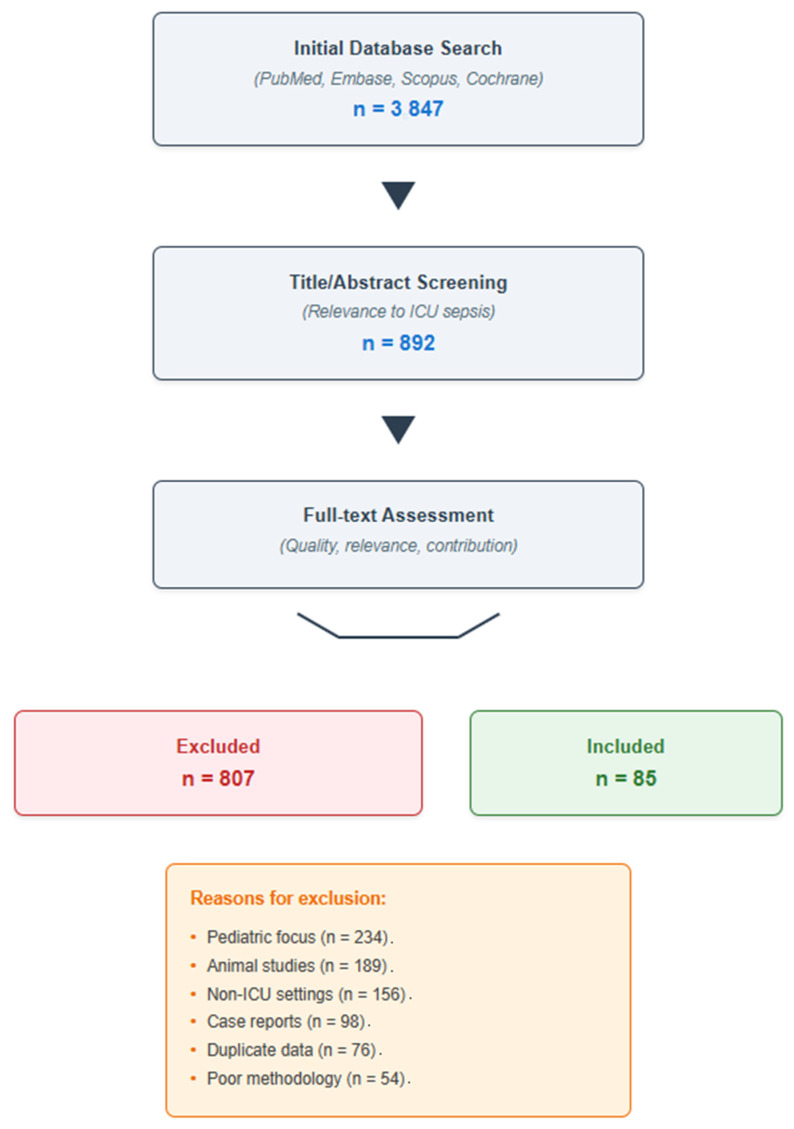
Literature selection process for this narrative review.

The initial search yielded 3847 articles (Figure 1). After title and abstract screening for relevance to ICU sepsis management, 892 articles underwent full-text assessment. Of these, 85 articles were ultimately cited in this review, selected based on their scientific rigor, clinical relevance, and contribution to understanding sepsis burden in ICU settings. Priority was given to systematic reviews, meta-analyses, large multicenter studies, and recent clinical guidelines. The selection process, while comprehensive, was not exhaustive, reflecting the narrative nature of this review.

## 3. Burden of Sepsis and Septic Shock in the ICU: Incidence, Prevalence, and Outcomes

Sepsis and septic shock represent enduring challenges in ICUs, characterized by high incidence and alarming mortality despite evolving treatment paradigms. According to the GBD study for 2017, there were approximately 48.9 million incident sepsis cases globally, resulting in an estimated 11 million deaths—accounting for nearly one-fifth of all global mortality [8]. These figures underscore the magnitude of sepsis as a public health crisis. Focusing on the ICU setting, an updated systematic review and meta-analysis by Fleischmann-Struzek et al. estimated that the incidence of hospital-treated sepsis was around 189 per 100,000 person-years, with a pooled mortality rate of approximately 26.7% [9]. The severity increases further when considering ICU-managed sepsis, with hospital discharge mortality rates approaching 41.9% in this high-risk population. Septic shock, the most critical subset of sepsis, exacts an even greater toll. Vincent et al. conducted a comprehensive meta-analysis, reporting that septic shock incidence at ICU admission was 10.4% (95% CI, 5.9–16.1%), while incidence during the ICU stay was 8.3% (95% CI, 6.1–10.7%) [10]. Importantly, pooled ICU mortality among septic shock patients was around 37.3% (95% CI, 31.5–43.5%), and hospital mortality rose slightly to 39.0% (95% CI, 34.4–43.9%) [11]. When examining outcomes at 30 and 90 days, a meta-analysis spanning Europe, North America, and Australia revealed that 30-day mortality for septic shock averaged 34.7% (95% CI, 32.6–36.9%), increasing to 38.5% (95% CI, 35.4–41.5%) at 90 days [12]. Regional analyses highlighted modest geographic disparities—with 30-day septic shock mortality at 33.7% in North America, at 32.5% in Europe, and, notably lower, at 26.4% in Australia (95% CI, 18.1–34.6%) [12]. These findings not only reflect differences in patient demographics and resources but also variances in diagnostic criteria and care processes. Beyond these high-income contexts, LMICs face a more pronounced burden. For instance, Mekuria et al. referenced WHO estimates suggesting over 24 million new cases of septic shock annually, with disproportionate impact on LMICs where mortality remains elevated [13]. Adding a perspective from healthcare resource utilization, Paoli et al. estimated that sepsis imposes a significant economic burden in the United States, driven by longer hospital stays and intensive resource consumption—though specific ICU-related costs were not detailed in that analysis [14]. Despite regional improvements and guideline-based care, ICU mortality for sepsis and septic shock remains distressingly high across global settings. The modest decline in early mortality (e.g., between 2009 and 2011) has plateaued since, and heterogeneity in outcomes persists across geographies and care settings [12]. Contributing factors include variability in diagnostic definitions (e.g., Sepsis-2 vs. Sepsis-3), inconsistent implementation of evidence-based bundles, and disparities in healthcare infrastructure and staffing.

## 4. Pathophysiology and Clinical Spectrum

**Figure 2:**
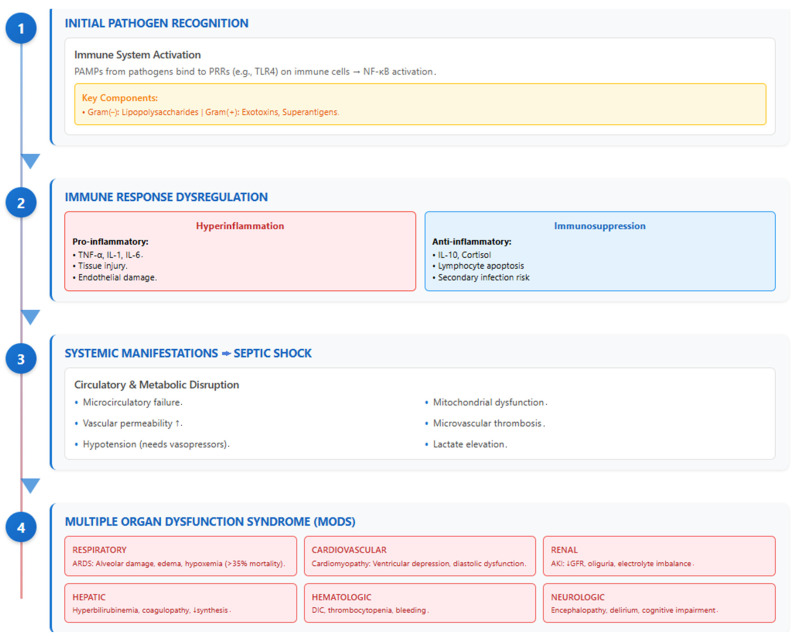
Pathophysiology of sepsis—from immune activation to multi-organ dysfunction.

Understanding sepsis and septic shock requires an appreciation of a dysregulated host immune response that transitions from localized pathogen defense to systemic inflammation, coagulation abnormalities, and, ultimately, multi-organ failure (Figure 2) [15].

Sepsis begins when the immune system encounters pathogen-associated molecular patterns (PAMPs), which bind to pattern recognition receptors (PRRs) like toll-like receptor 4 (TLR4). This interaction activates nuclear factor κB (NF-κB), leading to the release of key pro-inflammatory mediators, such as TNF-α, IL-1, and IL-6 [15]. While essential for pathogen control, this inflammatory cascade inadvertently contributes to tissue injury. The host also activates anti-inflammatory mediators (e.g., IL-10, cortisol), which, if overly robust, may impair the immune response—resulting in immunosuppression and predisposition to secondary infections [6]. Progression to septic shock reflects an escalation involving profound circulatory, cellular, and metabolic disruptions, such as persistent hypotension requiring vasopressors and elevated lactate levels. Mortality significantly increases at this stage, illustrating the critical need for early intervention [6,16].

The systemic immune dysregulation in sepsis typically impacts multiple organ systems. Sepsis-induced acute respiratory distress syndrome (ARDS) manifests through diffuse alveolar damage, leading to pulmonary edema, hypoxemia, and decreased lung compliance [17]. Mortality for ARDS remains high, often exceeding 35%, and survivors may suffer long-term cognitive and functional impairment [17]. Sepsis also provokes multiple organ dysfunction syndrome (MODS), driven by microcirculatory failure and mitochondrial dysfunction, which impair cellular oxygen utilization even when perfusion appears intact [18]. In the cardiovascular system, for instance, septic cardiomyopathy entails ventricular depression—particularly affecting diastolic filling—mediated by inflammatory cytokines and nitric oxide [11]. Hepatic failure, acute kidney injury, coagulopathy, and encephalopathy can similarly emerge in a predictable yet variable sequence, compounding patient mortality. The interplay between host and pathogen is a major driver of sepsis severity. Microbial factors, such as lipopolysaccharides from Gram-negative bacteria or exotoxins and superantigens from Gram-positive organisms, provoke intense immune activation [19]. Endothelial injury fosters increased permeability, microvascular thrombosis, and organ ischemia. Platelets amplify these effects by interacting with leukocytes and endothelium, promoting thrombosis and inflammation. Concurrently, immunological paralysis may ensue, characterized by lymphocyte apoptosis and suppressed adaptive immune responses [15,19].

## 5. Diagnostic Challenges and Biomarkers in ICU Sepsis

In the ICU, sepsis is a life-threatening and frequently time-critical diagnosis to make, which is made more difficult by the varied clinical phenotype of patients with sepsis and overlap with other forms of critical illness, as well as the fact that no single diagnostic test can be considered pathognomonic. Time until identification of the pathogen is critical since therapeutic windows are short, and delays in beginning antimicrobial therapy have been well established as associated with higher mortality rates [20]. The use of Sepsis-3 criteria, however, remains variable in real-world ICU practice, with significant delays in the identification of a large number of patients (Table 1) [16,21].

### 5.1. Bedside Assessment, Laboratory, and Imaging

Numerous bedside tools have been suggested to aid in identifying and supporting sepsis, each with distinct advantages and limitations. The quick SOFA (qSOFA) score was developed as a rapid screening tool for use outside the ICU, utilizing three simple clinical criteria: altered mental status (Glasgow Coma Scale < 15), systolic blood pressure ≤ 100 mmHg, and respiratory rate ≥ 22/min [22]. While qSOFA offers the advantage of bedside calculation without laboratory tests, its primary limitation lies in its poor sensitivity (approximately 60%) for detecting sepsis in ICU populations, where patients often already manifest organ dysfunction, making it unsuitable as a screening tool in critical care settings [22]. In contrast, the full SOFA score evaluates six organ systems (respiratory, coagulation, hepatic, cardiovascular, neurological, and renal) and demonstrates superior prognostic accuracy with better discrimination for mortality prediction (AUROC 0.74 vs. 0.66 for qSOFA) [21]. However, SOFA’s requirement for comprehensive laboratory testing, including arterial blood gases, bilirubin, creatinine, and platelet counts, can delay initial assessment by 30–60 min in unstable patients requiring immediate intervention [21]. Furthermore, while SOFA excels at quantifying organ dysfunction severity and tracking clinical trajectory, it was designed as an organ failure assessment tool rather than a sepsis diagnostic instrument, potentially leading to false positives in non-infectious critical illness [23]. Other tools, such as the National Early Warning Score (NEWS) and Modified Early Warning Score (MEWS), have shown utility in ward-to-ICU triage, yet their integration into ICU workflows remains inconsistent [23]. In ICU populations, routine laboratory markers (leukocyte count, C-reactive protein, and erythrocyte sedimentation rate) lack specificity due to systemic inflammation from trauma, burns, or postoperative states [24]. Similarly, lactate measurement serves as a valuable prognostic indicator and tissue hypoperfusion marker but may be elevated in non-septic conditions, such as seizures, hepatic failure, or beta-agonist use [25]. Imaging modalities (CT, ultrasound, PET-CT) are invaluable for identifying infection sources but may be limited by patient instability, transportation risks, and resource availability, particularly in LMIC ICUs [13].

### 5.2. Role of Microbiological Diagnosis and Emerging Biomarkers

Microbiological confirmation from blood cultures is the diagnostic gold standard for determining causative pathogens, optimal therapy, and, consequently, reducing unwarranted exposure to antimicrobials. Nonetheless, the amounts of clinical sample-positive cultures in ICU sepsis (30–50%) are unreliable and often take from 24 to 72 h to perform [26]. False negatives arising from antibiotic exposure, low pathogen burden, or false positives due to the presence of contaminants can lead to inappropriate therapy with important repercussions. Faster results and broader pathogen detection could be achieved by molecular techniques (multiplex PCR panels, next-generation sequencing), but the increased costs, lack of availability, and uncertain effects on hard outcomes in ICU practice remain relevant [27].

Efforts have therefore been directed to identify better biomarkers for the diagnosis, risk stratification, and treatment monitoring of sepsis over the last decade. Among these, procalcitonin (PCT) is the best studied; it increases with bacterial infections and correlates with disease severity, although PCT levels can also increase secondary to non-infectious systemic inflammation, such as major surgery or trauma [28]. Truly, PCT-guided antibiotic stewardship has been demonstrated to reduce antimicrobial treatment duration without an increase in mortality based on randomized controlled trials; however, its diagnostic specificity is still imperfect [29].

Mid-regional pro-adrenomedullin (MR-proADM) is currently a promising biomarker of endothelial activation and microcirculatory dysfunction, with better prognostic value compared to PCT and/or CRP in certain ICU cohorts [30]. Presepsin (soluble CD14 subtype) has emerged as a particularly valuable biomarker for both early diagnosis and mortality risk stratification. First described in multicenter studies [31], presepsin demonstrates faster kinetics than conventional biomarkers, rising within 2–3 h of sepsis onset compared to 6–12 h for PCT and 12–24 h for CRP [31]. This rapid elevation enables earlier sepsis detection and timely intervention. Meta-analyses have shown that elevated presepsin levels at presentation strongly predict adverse outcomes, with levels > 864 pg/mL demonstrating pooled sensitivity of 0.76 and specificity of 0.77 for 30-day mortality, outperforming PCT for prognostic assessment [32]. Prospective studies have confirmed that presepsin levels > 1200 pg/mL independently associate with in-hospital mortality (adjusted OR 3.45, 95% CI 2.12–5.61), supporting its use as a complementary tool to PCT and CRP for comprehensive sepsis assessment [33]. Other biological markers, such as soluble triggering receptor expressed on myeloid cells-1 (sTREM-1), have demonstrated utility for early discrimination of sepsis from non-infectious systemic inflammatory response syndrome (SIRS), though their performance remains inconsistent across studies [19,34].

New possibilities to phenotype septic patients more accurately are opening through omics-based approaches (transcriptomics, proteomics, and metabolomics) [35]. In ICU populations, gene expression panels (e.g., the Sepsis MetaScore and SeptiCyteLAB) exhibit high specificity for differentiating sepsis from sterile inflammation but remain largely unavailable in non-research settings [31].

### 5.3. Point-of-Care and AI-Driven Tools

Point-of-care testing (POCT) platforms capable of delivering rapid biomarker results at the bedside are increasingly being integrated into clinical workflows. These systems shorten turnaround times, facilitating earlier escalation or de-escalation of therapy. However, validation in diverse clinical environments remains incomplete, and cost-effectiveness analyses are needed before widespread adoption [36].

Artificial intelligence (AI) and machine learning (ML) models for sepsis prediction have shown promise, though their clinical impact depends critically on the setting of deployment. While most development has focused on ICU populations due to data availability (MIMIC-IV, eICU, Amsterdam, HiRID datasets), the clinical yield of ICU-centric prediction tools is inherently limited—most ICU admissions already involve suspected sepsis, leaving minimal actionable lead time for intervention [37]. The NAVOY^®^ Sepsis trial exemplified this challenge: despite achieving accurate prediction (~3 h before onset) in ICU patients, the randomized controlled trial showed no improvement in patient-centered outcomes, including ICU/hospital length of stay or mortality, illustrating the gap between statistical performance (AUROC) and clinical benefit [38]. In contrast, ward and emergency department settings offer greater opportunity for impact. A recent stepped-wedge cluster randomized trial of a qSOFA-based e-alert system on general wards demonstrated significantly lower 90-day in-hospital mortality with electronic screening versus standard care, providing the strongest causal evidence to date supporting early detection outside the ICU [39]. Similarly, while the TREWScore algorithm was initially developed using ICU data, its subsequent deployment focused on earlier care settings, though observational studies have raised methodological concerns about selection and immortal time biases that may overestimate treatment effects [40,41,42].

Future AI/ML development should prioritize emergency departments and general wards where early identification can meaningfully alter management trajectories, rather than adding more ICU-only prediction models with limited clinical utility. Researchers working exclusively with open ICU datasets should recognize that models developed in these data-rich but intervention-poor settings may have minimal real-world impact. Successful implementation requires prospective validation with hard clinical outcomes, integration with workflow-anchored alerts, and careful attention to the timing of interventions relative to disease progression. The translation of diagnostic innovations into improved outcomes faces multiple barriers: in high-income countries, integration into complex clinical workflows and clinician adherence to alert-based systems remain challenges, while in LMICs, the primary obstacles are infrastructural—limited laboratory capacity, scarcity of POCT devices, and minimal access to advanced diagnostics [13]. Future strategies should focus on multi-modal diagnostic pathways that combine clinical scoring, rapid pathogen detection, and host-response biomarkers into integrated decision-support platforms tailored to the specific care setting and available resources [43].

## 6. Advanced Management Strategies for Sepsis and Septic Shock in the ICU

### 6.1. Early Resuscitation and Hemodynamic Optimization

**Figure 3:**
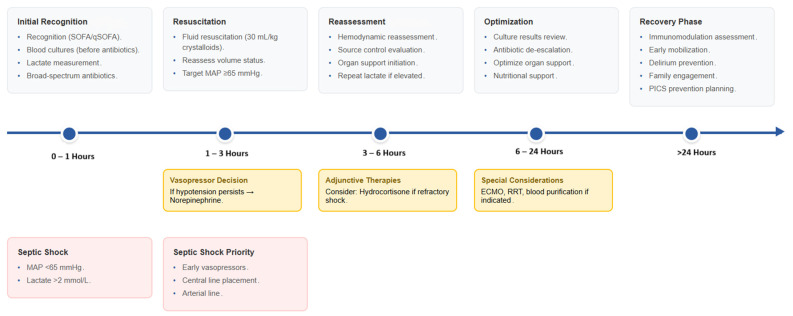
Integrated management timeline for sepsis and septic shock in the ICU.

The management of sepsis and septic shock in the ICU requires a multifaceted, time-sensitive, and protocol-driven approach aimed at rapid stabilization, infection control, and mitigation of organ dysfunction (Figure 3). While the principles of early recognition and timely intervention are universally endorsed, their practical execution must be adapted to patient-specific physiology, local microbiological epidemiology, and resource availability [44]. Prompt hemodynamic resuscitation remains central to reversing septic shock-induced tissue hypoperfusion. The 2021 Surviving Sepsis Campaign (SSC) guidelines maintain the recommendation for initial fluid resuscitation with 30 mL/kg of crystalloids for patients with sepsis-induced hypoperfusion, though this approach has been increasingly questioned following recent evidence [45]. The CLOVERS and CLASSIC trials (2022–2023) demonstrated that restrictive fluid strategies (prioritizing early vasopressor use with limited fluid administration) achieved similar 90-day mortality outcomes compared to liberal fluid resuscitation, while potentially reducing fluid overload complications [46,47]. Consequently, current practice is evolving toward more individualized fluid management, with the 30 mL/kg recommendation serving as a starting point rather than a rigid target, particularly in patients with pre-existing cardiac dysfunction or acute respiratory distress syndrome [48].

Point-of-care ultrasound (POCUS) has emerged as an invaluable tool for guiding resuscitation, enabling real-time assessment of cardiac function, volume status through inferior vena cava collapsibility, lung water via B-lines quantification, and identification of occult infection sources [49]. The FALLS protocol (Fluid Administration Limited by Lung Sonography) exemplifies POCUS integration, using progressive lung ultrasound findings to titrate fluid therapy and identify the optimal transition point to vasopressor support [50]. Balanced crystalloids, such as lactated Ringer’s or Plasma-Lyte, remain preferred over normal saline based on evidence of reduced hyperchloremic acidosis, acute kidney injury, and mortality [51]. For adults with sepsis or septic shock requiring large volumes of crystalloids, the SSC guidelines suggest considering albumin in addition to crystalloids, based on evidence of potential hemodynamic benefits and reduced net fluid balance, though this remains a weak recommendation with moderate-quality evidence [45]. Dynamic indices demonstrate superior predictive value compared to static measures (e.g., central venous pressure) for assessing fluid responsiveness. These include pulse pressure variation, stroke volume variation, and passive leg-raising tests coupled with cardiac output monitoring. However, their reliability diminishes in spontaneously breathing patients and those with arrhythmias [52]. The concept of “fluid tolerance” rather than fluid responsiveness is gaining traction, recognizing that the ability to increase cardiac output with fluids does not necessarily indicate that fluids will improve patient outcomes [53]. Vasopressors are indicated for persistent hypotension despite initial fluid resuscitation, with norepinephrine remaining the first-line agent targeting a mean arterial pressure of 65 mmHg [45]. Early vasopressor initiation, even during ongoing fluid resuscitation, may prevent excessive fluid accumulation and is increasingly advocated [54]. Vasopressin (up to 0.03 units/min) or epinephrine may be added in refractory cases, while dopamine use is discouraged except in highly selected bradycardic patients with low risk of tachyarrhythmias [45]. Angiotensin II has emerged as a rescue therapy for catecholamine-resistant septic shock, demonstrating efficacy in raising mean arterial pressure and potentially improving renal function, though definitive survival benefits await confirmation in larger trials [55].

### 6.2. Antimicrobial Therapy: Timing, Spectrum, and Stewardship

Timely initiation of effective antimicrobial therapy remains one of the strongest predictors of survival in septic shock [20,56]. The SSC advises administration within one hour of recognition for patients with septic shock and within three hours for sepsis without shock [48]. Empirical regimens should be guided by suspected infection source, local resistance patterns, and patient risk factors for multidrug-resistant organisms [57].

Combination therapy (e.g., beta-lactam plus aminoglycoside or fluoroquinolone) is considered in severe presentations or when Pseudomonas aeruginosa or other highly resistant pathogens are possible, although meta-analyses suggest mortality benefit is limited to the sickest patients. Antimicrobial de-escalation, based on culture results and clinical trajectory, is critical to reduce selective pressure and prevent Clostridioides difficile infection [58].

### 6.3. Source Control and Adjunctive Therapies

Source control—defined as physical measures to eliminate the focus of infection and control ongoing contamination—is a cornerstone of sepsis management. Examples include abscess drainage, infected catheter removal, biliary decompression, and debridement of necrotic tissue. Delays beyond 6–12 h after diagnosis have been associated with significantly increased mortality [59]. In the ICU, the decision-making process must balance the urgency of intervention against procedural risks in hemodynamically unstable patients. Corticosteroids, specifically intravenous hydrocortisone (200 mg/day), are recommended in patients with septic shock unresponsive to fluids and vasopressors, as they may accelerate shock reversal and reduce vasopressor dependence, though survival benefit remains inconsistent [45,60]. Blood purification strategies—including hemoadsorption devices to remove circulating endotoxins and cytokines—have generated interest but lack robust evidence of improved outcomes in large randomized trials [61]. Intravenous immunoglobulins (IVIGs), particularly IgM-enriched formulations, have shown potential benefit in certain subgroups (e.g., toxic shock syndrome), yet guideline recommendations remain cautious pending confirmatory evidence [62].

### 6.4. Mechanical Ventilation, Organ Support, and Immunomodulation

In patients with sepsis-induced ARDS, lung-protective ventilation—tidal volumes of 6 mL/kg predicted body weight and plateau pressures < 30 cm H_2_O—is the standard of care [17,63]. Prone positioning improves oxygenation and reduces mortality in moderate-to-severe ARDS, and it should be implemented early where feasible [64]. Renal replacement therapy (RRT) is indicated for severe acute kidney injury with complications such as refractory hyperkalemia, acidosis, or volume overload. Continuous modalities (CRRT) are preferred in hemodynamically unstable patients, although meta-analyses show no clear mortality advantage over intermittent hemodialysis [65]. Extracorporeal membrane oxygenation (ECMO), including awake strategies, may be considered as rescue therapy in refractory hypoxemia or circulatory collapse, typically in specialized centers with experienced multidisciplinary teams [66]. The immunopathology of sepsis spans an early hyper-inflammatory phase and a later immunosuppressive state [15,33]. Precision immunomodulatory strategies aim to tailor therapy to the patient’s immune phenotype. Trials of immune checkpoint inhibitors (e.g., anti-PD-1, anti-PD-L1) in immunosuppressed septic patients are ongoing, while agents targeting excessive inflammation (e.g., IL-6 receptor antagonists) are under investigation [67]. Mesenchymal stem cell therapy, leveraging their immunoregulatory and tissue-repair capacities, has shown safety and immunomodulatory efficacy in early-phase studies, though clinical benefit remains unconfirmed [68].

### 6.5. Blood Purification and Hemoperfusion Therapies: Evidence and Patient Selection

Blood purification strategies targeting endotoxin removal and cytokine modulation have shown promise in selected sepsis phenotypes, though translation to consistent mortality benefit remains elusive. The evidence base reveals a complex landscape where patient selection, timing, and specific endpoints critically influence therapeutic success. Endotoxin-targeted therapies have demonstrated the most consistent benefits in Gram-negative septic shock, particularly with intra-abdominal sources. The EUPHAS trial showed polymyxin-B hemoperfusion reduced 28-day mortality from 53% to 32% (*p* = 0.03) and improved hemodynamics in severe abdominal septic shock [69]. However, the larger EUPHRATES trial failed to demonstrate overall mortality benefit, though post hoc analysis revealed potential efficacy in patients with endotoxin activity assay (EAA) levels between 0.60 and 0.89, suggesting the critical importance of biomarker-guided patient selection [70,71]. More recently, the Efferon LPS cartridge demonstrated faster shock resolution and reduced early mortality (day 3) but no difference at 14 or 28 days, highlighting the distinction between hemodynamic improvement and sustained survival benefit [72]. Cytokine adsorption technologies, including CytoSorb, have consistently shown effective cytokine reduction and hemodynamic stabilization without translating to mortality benefit in randomized trials [73]. The heterogeneity in outcomes across different blood purification modalities suggests that benefit may be restricted to specific phenotypes: patients with documented endotoxemia (EAA > 0.6), intra-abdominal infection sources, or extreme cytokine elevation initiated within 24 h of shock onset. Current evidence positions these therapies as potential adjuncts for shock reversal and vasopressor reduction rather than primary mortality-reducing interventions, with ongoing trials focusing on enriched populations and combined biomarker-device strategies to better define their therapeutic niche [74].

### 6.6. Bundled Care and Systems-Level Implementation

Structured care bundles—such as the SSC’s Hour-1 Bundle—integrate early recognition, timely antimicrobials, fluid resuscitation, hemodynamic reassessment, and lactate monitoring [45]. Adherence to bundles is consistently associated with improved outcomes, yet implementation varies widely, with adherence rates often below 50% in real-world ICU audits [75]. Barriers include staffing constraints, delayed laboratory results, and resistance to protocolization in complex cases.

Quality improvement initiatives, including real-time performance feedback, sepsis champions, and electronic health record-based alerts, have demonstrated sustained improvements in compliance and survival [76]. In patients with advanced comorbidities, poor functional status, or refractory multi-organ failure, discussions about goals of care and transition to comfort-focused management are essential. Early involvement of palliative care teams in the ICU can improve symptom control, patient–family satisfaction, and alignment of care with patient values [77].

## 7. Long-Term Outcomes and Post-Intensive Care Sequelae in Sepsis Survivors

Recognition, resuscitation bundles, and critical care advances in early identification have led to improved sepsis- and septic shock-related outcomes in patients admitted to the ICU. Nevertheless, the expanding registry of survivors bears a significant burden from the myriad of persistent physical, cognitive, and psychological abnormalities encompassed by the syndrome known as post-intensive care syndrome (PICS) [78]. The associated sequelae can be long-lasting and lead to a substantial reduction in quality of life, functional independence, and socioeconomic stability.

### 7.1. Mortality and Physical Sequelae

Sepsis survivors face elevated mortality risk after hospital discharge. Follow-up studies show that 30–50% of individuals die within one or two years after hospitalization for pneumonia (most commonly due to recurrent infections, cardiovascular events, or progressive frailty [79]). Older age, pre-existing comorbidities, protracted ICU stay, or severe multi-organ dysfunction make patients at risk for long-term mortality [80]. In survivors, ongoing immune dysregulation in the form of lymphopenia and abnormal cytokine profiles that continue for months after discharge could place them at higher risk of secondary infections and cancer relapse [81]. Critical illness polyneuropathy (CIP) and critical illness myopathy (CIM) represent a frequent component of the neuromuscular disorders that occur in sepsis survivors, with an incidence listed up to 46% in long-stay ICU patients [82]. These are characterized by muscle weakness, poor mobility, and exercise endurance, which may last for years. Immobilization and nutritional deficits associated with catabolic inflammation contribute to sepsis-induced sarcopenia, which impedes full functional recovery [83,84].

### 7.2. Cognitive Impairment and Psychological Impact

ICU-acquired cognitive dysfunction is prevalent in sepsis survivors, with deficits in memory, attention, processing speed, and executive function reported in up to 62% of patients at three months post-discharge [85]. These disabilities may last for years, leading to lower rates of returning to work and increased dependency on activities of daily living. The pathophysiology is multifactorial: systemic inflammation, microvascular injury, blood–brain barrier disruption, and hypoperfusion contribute to neuronal injury and neurodegeneration [86]. Sepsis survivors show hippocampal atrophy and white matter lesions, which are consistent with neurocognitive decline detected by neuroimaging studies [87]. PICS comprises a significant psychiatric morbidity scenario. Depressive disorders, anxiety disorders, and posttraumatic stress disorder (PTSD) are seen in 20–40% of sepsis survivors, with symptoms often appearing weeks to months after hospital discharge [88]. Traumatic ICU memories, lack of sleep, and delirium are aggravating factors, as well as social isolation after they recover. Psychological symptoms impair compliance with rehabilitation programs, which impedes physical recovery [89].

### 7.3. Post-Sepsis Syndrome and Immunological Legacy

A distinct entity—post-sepsis syndrome—has been described, with recurrent infections, slow wound healing, and chronic inflammation [84]. This overactive immune response leads to accumulation of suppressive myeloid-derived cells, decreased antigen presentation, and, ultimately, functional exhaustion of T cell and NK cell responses—persistent immune dysregulation, which is challenging to reverse [90]. These changes could persist for months, leading to an extended period of susceptibility. Chronic low-grade inflammation has also been associated with an increased risk of atherosclerosis and cardiovascular events in sepsis survivors, irrespective of traditional risk factors [91].

### 7.4. Socioeconomic Burden and Follow-Up Strategies

Sepsis involves long-term sequelae that also affect social and economic aspects. Survivors often suffer from decreased work ability or disability and therefore lose income and become more dependent on social services [92]. Family caregivers assume caregiving can be emotionally taxing, causing financial distress and resulting in compromise of their health, known as caregiver burden [93,94]. Early mobilization in the ICU with structured physical rehabilitation programs has been shown to enhance functional outcomes and decrease long-term disability [19]. An emerging model for comprehensive follow-up is through multidisciplinary post-ICU clinics with critical care providers, rehabilitation specialists, psychologists, and social workers [95]. There is no reason why screening for cognitive impairment and mental health disorders should not form part of standard post-sepsis care pathways, with appropriate referral to neuropsychology or psychiatry services as required [96]. Sarcopenia and muscle atrophy are caused by the acute phase response, which can be reversed by optimal delivery of nutrition in conjunction with resistance exercise training [83]. In particular, telemedicine-based follow-up could represent a high-volume solution to close access gaps, especially in resource-limited settings [97].

### 7.5. Research Gaps and Future Directions

While recognition of PICS and post-sepsis syndrome has grown, high-quality interventional trials targeting these conditions are scarce. Key research priorities include identifying biomarkers to predict long-term outcomes, defining optimal rehabilitation timing and intensity, and developing targeted therapies to reverse persistent immune dysfunction [90,98]. Health policy initiatives must address the continuity of care from ICU discharge to community reintegration, ensuring that survivors receive structured follow-up, rehabilitation, and psychosocial support. Integrating survivorship care into national sepsis action plans could improve long-term outcomes and reduce readmissions.

## 8. Conclusions

Sepsis and septic shock continue to be formidable challenges in intensive care medicine, with acute medical consequences for the individual patient but also profound longer-term health burdens that have societal and economic impacts. Despite nearly a half-century of investigation and advances in early identification, antimicrobial therapy, hemodynamic support, and organ-protective strategies, mortality remains unacceptably great, and survival is plagued by persistent morbidity. The pathophysiological enigma of sepsis, encompassing as it does a composite cross-talk between excessive inflammation, immune paralysis, microvascular failure, and metabolic anomalies, remains an equally versatile opponent that is difficult to overcome by off-the-shelf therapeutic tools. Adding to the challenge is the variability in patients, infections, comorbidities, and geographical contexts, with the latter particularly relevant between high-income countries and lower and upper LMICs. Beyond the potential for efficiency, bundled protocols, such as those prescribed by the SSC guidelines, have demonstrated lifesaving efficacy if implemented successfully, though international adoption has been variable, and uptake of trial evidence into day-to-day practice is typically slow.

## Figures and Tables

**Table 1:** Evolution of sepsis definitions and diagnostic criteria.

Criteria	Sepsis-1 (1991)	Sepsis-2 (2001)	Sepsis-3 (2016)
Definition	Systemic inflammatory response syndrome (SIRS) in response to infection	Documented or suspected infection plus systemic inflammatory response	Life-threatening organ dysfunction caused by a dysregulated host response to infection
Key Criteria	SIRS criteria, ≥2: Temperature >38 °C or <36 °C. Heart rate > 90 bpm. Respiratory rate > 20/min or PaCO_2_ < 32 mmHg. WBC > 12,000 or <4000/mm^3^ or >10% bands.	Same as Sepsis-1 with expanded list of possible signs, including the following: Altered mental status. Hyperglycemia (>140 mg/dL) in absence of diabetes. Arterial hypotension. Elevated lactate.	Suspected infection AND the following: Acute increase in SOFA score ≥ 2 points. Septic shock: sepsis with hypotension requiring vasopressors to maintain MAP ≥ 65 mmHg AND lactate > 2 mmol/L despite adequate fluid resuscitation.
Clinical Tools	SIRS criteria (primary tool)	SIRS criteria. Expanded diagnostic criteria list. Early goal-directed therapy protocols.	SOFA score (ICU). qSOFA score (non-ICU): - Altered mental status. - SBP ≤ 100 mmHg. - RR ≥ 22/min.
Limitations	Poor specificity. SIRS present in many non-infectious conditions. Did not predict outcomes well. Overdiagnosis of sepsis.	Still relies heavily on SIRS. Complex criteria difficult to apply clinically. Does not improve outcome prediction. Lack of clear organ dysfunction assessment.	SOFA requires full laboratory workup. qSOFA has low sensitivity (30–50%). May miss early sepsis. Less useful in immunocompromised patients. Requires baseline SOFA for comparison.
Mortality Implications	Hospital mortality ~16%. Wide variation based on severity. Poor discrimination between sepsis severities.	Sepsis: 10–20% mortality. Severe sepsis: 20–40% mortality. Septic shock: 40–70% mortality.	Sepsis: ~10% hospital mortality. Septic shock: 40% hospital mortality. Each SOFA point increase associated with ~10% increase in mortality.

SIRS, systemic inflammatory response syndrome; SOFA, Sequential Organ Failure Assessment; qSOFA, quick SOFA; WBC, white blood cell count; MAP, mean arterial pressure; SBP, systolic blood pressure; RR, respiratory rate; ICU, intensive care unit.

## Data Availability

No new data were created.

## Abbreviations

The following abbreviations are used in this manuscript:

CU	Intensive Care Unit
PICS	Post-Intensive Care Syndrome
MR-proADM	Mid-Regional Pro-Adrenomedullin
AI	Artificial Intelligence
GBD	Global Burden of Disease
LMICs	Low- and Middle-Income Countries
ARDS	Acute Respiratory Distress Syndrome
MODS	Multiple Organ Dysfunction Syndrome
PAMPs	Pathogen-Associated Molecular Patterns
PRRs	Pattern Recognition Receptors
TLR4	Toll-Like Receptor 4
NF-κB	Nuclear Factor κB
TNF-α	Tumor Necrosis Factor-α
IL-1	Interleukin-1
IL-6	Interleukin-6
IL-10	Interleukin-10
SIRS	Systemic Inflammatory Response Syndrome
SOFA	Sequential Organ Failure Assessment
qSOFA	Quick Sequential Organ Failure Assessment
NEWS	National Early Warning Score
MEWS	Modified Early Warning Score
CRP	C-Reactive Protein
ESR	Erythrocyte Sedimentation Rate
CT	Computed Tomography
PET-CT	Positron Emission Tomography-Computed Tomography
PCR	Polymerase Chain Reaction
PCT	Procalcitonin
sTREM-1	Soluble Triggering Receptor Expressed on Myeloid Cells-1
POCT	Point-Of-Care Testing
ML	Machine Learning
SSC	Surviving Sepsis Campaign
MAP	Mean Arterial Pressure
SBP	Systolic Blood Pressure
RR	Respiratory Rate
WBC	White Blood Cell
IVIG	Intravenous Immunoglobulin
IgM	Immunoglobulin M
RRT	Renal Replacement Therapy
CRRT	Continuous Renal Replacement Therapy
ECMO	Extracorporeal Membrane Oxygenation
PD-1	Programmed Cell Death-1
PD-L1	Programmed Cell Death-Ligand 1
CIP	Critical Illness Polyneuropathy
CIM	Critical Illness Myopathy
PTSD	Post-Traumatic Stress Disorder
NK	Natural Killer
EEG	Electroencephalogram
WHO	World Health Organization
